# Gamification: What It Is and Why It Matters to Digital Health Behavior Change Developers

**DOI:** 10.2196/games.3139

**Published:** 2013-12-12

**Authors:** Brian Cugelman

**Affiliations:** ^1^AlterSpark Corp.Toronto, ONCanada

**Keywords:** behavioral medicine, behaviour and behavior mechanisms, behavioral research, behavioral sciences, persuasive communication, health psychology, psychology, experimental game, interactive games, computer games

## Abstract

This editorial provides a behavioral science view on gamification and health behavior change, describes its principles and mechanisms, and reviews some of the evidence for its efficacy. Furthermore, this editorial explores the relation between gamification and behavior change frameworks used in the health sciences and shows how gamification principles are closely related to principles that have been proven to work in health behavior change technology. Finally, this editorial provides criteria that can be used to assess when gamification provides a potentially promising framework for digital health interventions.

## Introduction

Although health behavior change research suggests that it is easy to influence how people think and behave, practitioners who have worked in the health behavior change field, with populations or individuals, will often complain that that behavior change is difficult to achieve, expensive, and impacts are often short-lived.

The average public health campaign is able to impact the behavior of roughly 5% of a population [1], while a meta-analysis that I co-investigated a few years ago showed that online behavior change technologies could impact the behavior of roughly 10% of their users [2](this figure was derived by comparing a Pearson's coefficient effect size to a percentage, as used by Snyder (2007); however, this method is subject to significant statistical bias [3] and should therefore only be taken as a ballpark figure at best). Given the modest impacts from evidence-based interventions, why are we now witnessing widespread claims that gamification makes it easy to shape how people think and behave, simply by rewarding users with points and badges? Is gamification really a magic solution to shaping behavior, or simply, unrealistic hype?

In this editorial, I describe and evaluate gamification, address misconceptions, show linkages to health behavior change theory, and advocate when gamification is a good or bad approach for digital health behavior change interventions.

## Hype Around Gamification

At present, there is no shortage of gamification advocates who claim badges, points, and competition will get everyone so hooked on digital technologies, that developers should gamify their interventions immediately, or get left behind. However, jumping on this gamification bandwagon is a risky undertaking. Not because gamification does not work, but rather, because it is easy to get it wrong if developers do not understand what it is, know its limits, and make informed decisions on its application. Gamification is just one of many persuasive architectures. However, like all other persuasive design patterns, gamification has merit when used in the right way, under the right circumstances.

## The Active Ingredients of Gamification

Gamification is defined as the use of game design elements in non-game contexts [4]. The idea is that if we can isolate the active ingredients that make games addictive, then intervention developers can put those ingredients into their digital technologies and make them addictive too. For instance, we can make a routine non-game activity, such as taking medication, into a game that is fun and engaging by adding game elements, such as earning points for taking medications.

To apply gamification, developers first need a list of game design elements, and then second, they need to integrate these elements into their intervention. However, the problem is that gamification researchers do not always agree on what these ingredients are, and some researchers take the position that these ingredients cannot even be named.

Within this debate, I take the view that technology is only persuasive when it employs specific behavior change ingredients, as one of the key principles of evidence based behavioral medicine [5-7]. These persuasive ingredients are the factors that exert persuasive force on people, encouraging them to shift their beliefs, attitudes, and actions. If these ingredients are removed, the technology is no longer persuasive. In the sciences, these ingredients have different names, but I will refer to them as "behavior change strategies", "persuasive strategies", or simply, "strategies".

To identify these gamification strategies, I reviewed a number of popular gamification taxonomies from academic and non-academic sources by Charles Coonradt [8], Reeves and Read [9], Gabe Zichermann [10], and Marc Prensky [11]. I identified the common strategies listed by these authors, and compared them to a taxonomy of interactive behavior change and persuasive design strategies within my Persuasive Communication Model [12].

After, I identified 7 core ingredients of gamification that have clear linkages to proven behavior change strategies, with the exception of fun and playfulness, which has perhaps, not received much attention in the health behavior change literature. These 7 ingredients of gamification are listed in Textbox 1.

My goal was to identify the persuasive architecture of gamification, the essential strategies that combine to produce an effect greater than the sum of its parts. Put another way, the persuasive architecture of gamification is the combination of ingredients that make a product fun and engaging. Take away some of these core ingredients, and the product becomes dull. Add them back in, and the magic happens. A persuasive architecture is the optimal blend of persuasive strategies for a particular application [2].

Whereas the strategies in Textbox 1 are the broad principles that make gamification addictive, the gamification mechanics (or tactics) are the on-screen features that users interact with. For instance, the strategy of motivating a user by *comparing their progress with others* can be implemented with the gamification tactic of *showing the game leaders*. Textbox 2 shows 10 of the most popular gamification tactics [13].

One of the chief misconceptions about gamification is that any technology that employs game tactics will be more engaging. The problem with this thinking is that it mistakes superficial game tactics for deeper psychological strategies. For instance, it is risky to believe that badges will motivate users, without considering the persuasive strategies that the game tactics must satisfy, where a badge’s value comes from a community that places value on that badge, and where the badge’s value is further dependent on whether it transfers anything of value to the person. Offering game tactics that do not satisfy persuasive strategies is like cooking dinner for someone with ingredients (game tactics) they do not like (strategies).

**The persuasive architecture of gamification and its 7 persuasive strategies.**

1. Goal setting: Committing to achieve a goal
2. Capacity to overcome challenges: Growth, learning, and development
3. Providing feedback on performance: Receiving constant feedback through the experience
4. Reinforcement: Gaining rewards, avoiding punishments
5. Compare progress: Monitoring progress with self and others
6. Social connectivity: Interacting with other people
7. Fun and playfulness: Paying out an alternative reality

**Popular gamification tactics.**

1. Providing clear goals
2. Offering a challenge
3. Using levels (incremental challenges)
4. Allocating points
5. Showing progress
6. Providing feedback
7. Giving rewards
8. Providing badges for achievements
9. Showing the game leaders
10. Giving a story or theme

## The Efficacy of Gamification

### Overview

In order for gamification to be considered effective, gamified technology must outperform other design patterns, in terms of its ability to influence people's beliefs, attitudes, or behaviors. Moreover, to be considered effective, gamification must sustain these impacts over the long-term, and offer more than a short-term novelty effect.

However, the question that is rarely asked is whether there is evidence that shows gamification can influence how people think or behave? To answer this question, there are perhaps four stream of evidence that we can draw from. They include (1) anecdotal evidence, (2) research on the efficacy of gamification, (3) ingredients that have been proven to work, and (4) persuasive architecture that is related to proven theories.

### Anecdotal Evidence

Much of the hype around gamification seems to come from ad hoc anecdotal evidence, in the form of case studies and industry claims. Although highly unreliable, this body of ad hoc success stories has served to raise awareness of gamification concepts, and prompted researchers to take a closer look at gamification.

### Research on the Efficacy of Gamification

As research on gamification started to appear just before 2010, we recently reached the point where there were enough quality academic studies, that a team of researchers conducted a systematic review of the scientific literature [13]. In their publication, "Does Gamification Work?", the research team found evidence across numerous studies, that gamification can influence psychological and physical outcomes, meaning gamification can make a digital product more fun and engaging.

However, not all studies showed positive effects, and the impact seemed to vary according to the community, users, and product, with some users complaining that gamification was annoying. Additionally, there were far more studies in particular contexts, such as online learning, intra-organizational systems, and work environments, with the lack of studies from other domains possibly signaling that gamification may only work in contexts that already share a common persuasive architecture. Finally, the researchers raised one red flag, as they could not tell if the reported outcomes represented sustainable long-term impacts, or just short-term novelty effects.

### Ingredients That Have Been Proven to Work

From the point of view of evidence-based behavioral medicine, the only thing that would matter in gamification is whether it employs principles and tactics that have been scientifically proven to influence health outcomes.

To quickly assess the link between gamification and health behavior change, I conducted an exploratory comparison of the 7 ingredients of gamification to behavioral science principles that have been proven to work in digital health behavior change interventions, drawing on validated principles from my prior meta-analysis on the factors that make health behavior change technologies work [2].

I mapped 27 techniques and principles to the 7 gamification strategies. Table 1 shows the top two most effective and statistically significant behavior change principle and techniques.

This exploratory mapping demonstrates that there are some promising links between gamification principles and digital health behavior change science, with one gap that stood out, being no strong link to fun and playfulness in health behavior change approaches. Although gamification shows some clear links to health behavior change strategies and tactics, the technical mechanics used in health behavior change interventions can be radically different than those used in gamified technologies, even though they may appeal to similar psychological faculties.

**Table 1:** Gamification strategies and validated behavior change ingredients.

Gamification strategies	Validated behavior change ingredients [2]
1. Goal setting	Agree behavioral contract Goal setting (behavior)
2. Capacity to overcome challenges	Time management Action planning
3. Providing feedback on performance	Prompt self-monitoring of behavioral outcome Prompt self-monitoring of behavior
4. Reinforcement	Provide rewards contingent on successful behavior
5. Compare progress	Prompt self-monitoring of behavioral outcome Provide normative information about others’ behavior
6. Social connectivity	Social influences (norms) Plan social support/social change
7. Fun and playfulness	N/A

### Persuasive Architecture That is Related to Proven Theories

Beyond the direct empirical evidence, there is also theoretical support for gamification, as a framework that shares many strategies in common with other theories that have been proven to work in the health field.

The persuasive architecture of gamification shares elements in common with coaching, which relies on a coach's ability to foster team member motivation, employ strategies to help their team overcome opposition, provide support in building member's techniques, and help members build their character [14]. The architecture of gamification is also extremely close to the cybernetic variations of self-regulation theory, based on feedback loops, which cover all strategies except perhaps, social connectivity, and fun and playfulness [15]. Although gamification shares the same strategies, there are big differences in the tactical way that these strategies are implemented. However, the similarity does mean that it is easier to gamify digital interventions modeled on coaching or self-regulation theory, because they are already quite similar.

One of the theories that is infrequently used in the health field, but popular among video game designers, is *flow*, the study of how people become absorbed and engaged in an activity when they are doing something where their skill level is perfectly matched to the challenge level [16]. According to the principle of flow, if a game is too difficult, people will become stressed and stop playing. If a game is too easy, people will become bored and stop playing. But if the challenge keeps increasing as the person’s skill increases, they will have a flow experience, become absorbed in the task at hand, and experience a meditative-like absorption in what they are doing. Bringing people to this state of mind is a key goal in game design.

## Selecting the Right Persuasive Architecture for an Interventions

Although there is evidence that suggests gamification works, there are some major risks associated with the current gamification hype. The chief risk is becoming overconfident in the ability of gamification to exert massive influence across all contexts, which can cause developers to form tunnel vision and fixate on just one of many persuasive architectures.

Locking into one framework might cause developers to miss opportunities to identify the best architecture for the job. Every persuasive architecture has its own unique mix of ingredients, and suitability to particular users and contexts. For instance, a sign-up landing page, health screener, donation page, or social networking site all draw on different combinations of persuasive ingredients. Moreover, my recent research is showing that the world's most successful websites are hyper optimized, often offering more persuasive strategies per square inch than many of the less popular sites.

What matters in behavior change design is knowing which persuasive architecture is right for a particular application, and identifying when gamification, in whole or part, is suitable to a particular application.

## Assessing the Suitability of Gamification

Intervention developers should only use gamification when it is suitable to a given audience-product mix. However, it is not easy to know in advance whether or not gamification makes sense for a particular project and its unique audience-intervention mix.

To evaluate if gamification is suitable to a particular intervention, Textbox 3 presents criteria that developers can use to evaluate when gamification offers a promising framework. However, users are the ultimate judges of intervention efficacy, so any gamified interventions will require user testing, to determine if they can work or not.

**Criteria to consider when evaluating if gamification is suitable to a particular intervention.**

1. The intervention users
2. The users’ social context
3. The psychological and behavioral outcomes that are being pursued
4. How closely the intervention's logic model or theory of change fits with the persuasive architecture of gamification
5. The interactive product or platform that is being planned
6. The compatibility of the interactive product, users, and community with the 7 gamification strategies
7. The compatibility of the interactive product, users, and community with gamification tactics

## Final Thoughts

There is promising evidence that suggests gamification works, and on the surface, gamification appears to share elements in common with proven health behavior change approaches. Given this, it is easy to see how existing digital interventions can borrow gamification principles, by considering flow, meaningful rewards, making them more social, and most importantly, finding innovative ways to make digital health interventions fun and engaging.

JMIR Serious Games is a new important journal devoted to research and opinion around games and gamification for behavior change and other applications, and as one of the editorial board members I look forward to help building the evidence base in this emerging area.
